# Adherence to Mediterranean diet and risk of developing cognitive disorders: An updated systematic review and meta-analysis of prospective cohort studies

**DOI:** 10.1038/srep41317

**Published:** 2017-01-23

**Authors:** Lei Wu, Dali Sun

**Affiliations:** 1 Department of Epidemiology, Institute of Geriatrics, Chinese People’s Liberation Army General Hospital, Beijing, China; 2 Department of Nanomedicine, Houston Methodist Research Institute, Houston, United States

## Abstract

Recent articles have presented inconsistent findings on the impact of Mediterranean diet in the occurrence of cognitive disorders; therefore, we performed an updated systematic review and meta-analysis to evaluate the potential association and dose-response pattern with accumulating evidence. We searched the PubMed and the Embase for the records relevant to this topic. A generic inverse-variance method was used to pool the outcome data for continuous variable, and categories of high vs. low, median vs. low of Mediterranean diet score with a random-effects model. Generalized least-squares trend estimation model was used to estimate the potential dose-response patterns of Mediterranean diet score on incident cognitive disorders. We identified 9 cohort studies involving 34,168 participants. Compared with the lowest category, the pooled analysis showed that the highest Mediterranean diet score was inversely associated with the developing of cognitive disorders, and the pooled RR (95% CI) was 0.79 (0.70, 0.90). Mediterranean diet score of the median category was not significantly associated with cognitive disorders. Dose-response analysis indicated a trend of an approximately linear relationship of the Mediterranean diet score with the incident risk of cognitive disorders. Further studies of randomized controlled trials are warranted to confirm the observed association in different populations.

Cognitive disorders are responsible for an important public health issue with the aging of the population and the limited pharmacological therapies worldwide^1^,^2^. It is estimated that the total number of the elderly Americans with Alzheimer disease (AD) dementia is projected to be approximately 14 million in 2050^3^. Prevention of cognitive disorders is an urgent priority because of the difficulty of the modification of the course of age-related cognitive decline. Thus, it is important to identify its related modifiable lifestyle factors. Further effective and feasible strategies should be applied to slow down the progression of cognitive decline.

In recent decades, the interest of nutrients has shifted from the impact of single nutrients to the role of healthy dietary patterns, and the latter one is considered better reflects the complexity of our dietary behavior^4^. Mediterranean diet (characterizes by a high consumption of fruit, vegetables, legumes, cereals, and unsaturated fatty acids; low consumption of meat and saturated fatty acids; low to moderate consumption of dairy products; moderate to high consumption of fish; and a regular but moderate drinking of alcohol) is reported to have a protective effect of cognitive decline in a substantial number of qualitative researches^5^,^6^,^7^,^8^ and quantitative reviews^9^,^10^,^11^,^12^. Recently, 4 cohort studies have been published on this topic, and these high-quality articles included an additional 25,864 participants^13^,^14^,^15^,^16^. However, the findings of the 4 studies were inconsistent, and 2 of these studies observed a non-significant association between Mediterranean diet and cognitive disorders^13^,^15^. With the accumulating evidence, we therefore performed an updated systematic review and meta-analysis of prospective cohort studies to assess the influence of adherence to Mediterranean diet in the risk of developing cognitive disorders (including mild cognitive impairment, AD, and dementia). Furthermore, we also undertook a dose-response analysis to assess the potential dose-response patterns of Mediterranean diet score on the incident risk of cognitive disorders.

## Results

### Study Identification and Selection

**Figure 1:**
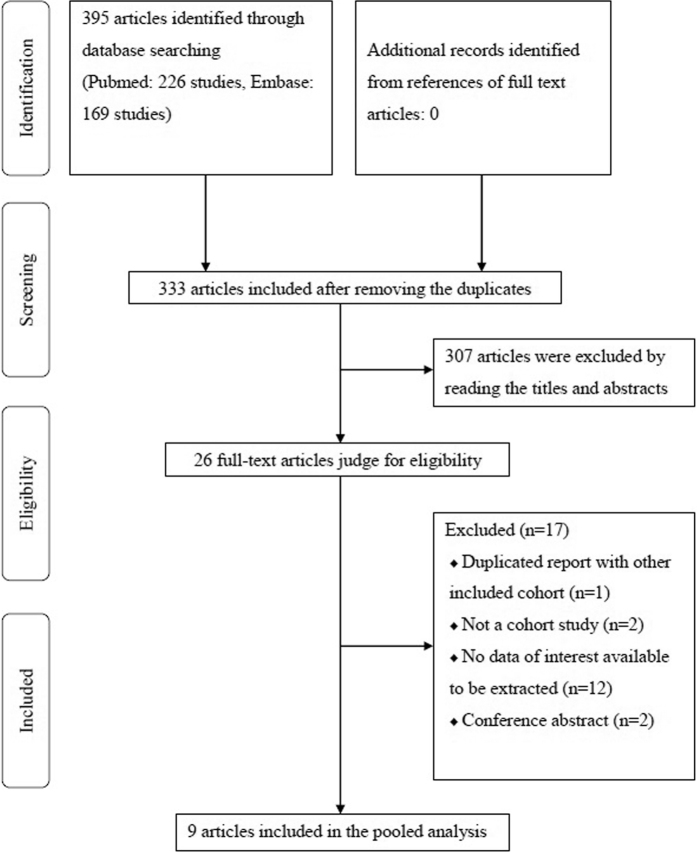
Flow diagram of the articles included in the present study.

Figure 1 shows the detailed flow diagram of articles included in the present study. A total of 395 articles were identified through the database searching (Pubmed: 226 studies, Embase: 169 studies). After removing the duplicates, 333 articles were remained for the further assessment. Next, 307 of the 333 articles were excluded after reading the titles and abstracts, and the remaining 26 full-text articles were estimated for eligibility. Finally, 9 articles were included in the present systematic review and meta-analysis^13^,^14^,^15^,^16^,^17^,^18^,^19^,^20^,^21^. Reasons for the exclusion of the 17 articles were listed in Supplementary Table 4.

### Study Characteristics

Table 1 shows the main characteristics of the included studies. The 9 articles were published between 2002 and 2016. Six of the included studies were performed in the United States^13^,^14^,^16^,^19^,^20^,^21^ and the other 3 studies were performed in Australia, France and Sweden, respectively^15^,^17^,^18^. The years of follow-up duration ranged between 2.2 and 12. Seven articles included both male and female participants^14^,^16^,^17^,^18^,^19^,^20^,^21^, and the other 2 articles included only male^13^ or only female participants^15^. The baseline age of the participants was 45 years or above. The number of participants ranged from 923 to 17,478 for a total number of 34,168. The Mediterranean diet was assessed by food-frequency questionnaire (FFQ) excepted for 2 articles^15^,^19^. Eight included studies used a same definition^22^ to define the adherence to the Mediterranean diet score (0–9 points); only 1 study^14^ used the definition by Panagiotakos *et al*.^23^ to define the Mediterranean diet score (0–55 points). The incident of cognitive disorders were diagnosed from DSM (Diagnostic and Statistical Manual of Mental Disorders)^13^,^15^,^18^ for dementia; NINCDS-ADRDA (National Institute of Neurological and Communicative Disorders and Stroke-Alzheimer’s Disease and Related Disorders Association) for AD^14^,^15^,^18^,^20^,^21^; MMSE (Mini-Mental State Examination) score^13^,^15^,^17^ or other standard criteria^16^,^19^,^21^ for mild cognitive impairment (MCI). All of the included studies followed a 9-point scale of Mediterranean diet score, except for 1 study^14^.

### Quality Assessment

All studies met the quality score of 7–9 stars (Supplementary Table 2). The main quality issues were listed as follows. Two articles did not use the FFQ to assess the Mediterranean diet score^15^,^19^. The follow-up duration was less than 5 years in 6 studies^14^,^16^,^18^,^19^,^20^,^21^. Diagnoses of MCI, AD, and Dementia were based on standard criterion in all articles. All included studies adjusted for potential confounding covariates.

### Mediterranean diet and cognitive disorders

**Figure 2:**
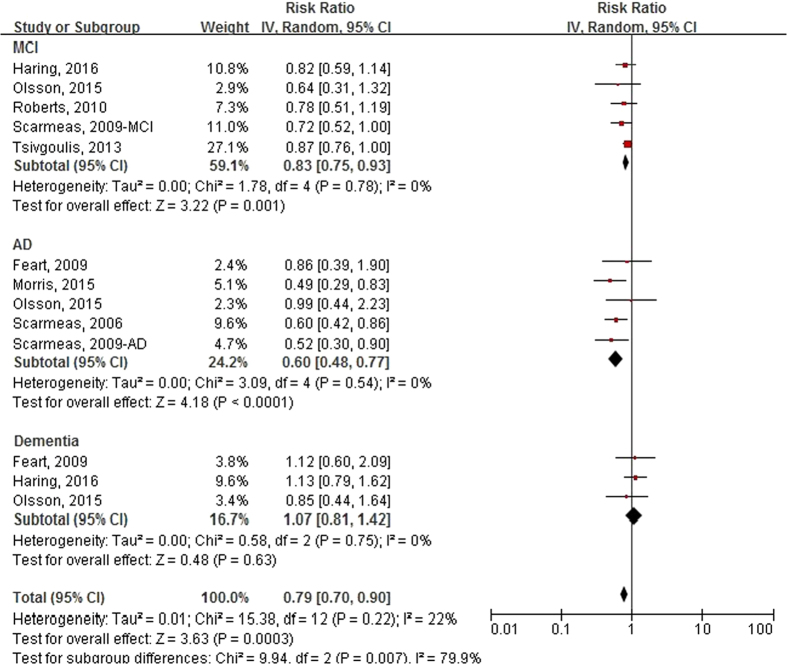
Forest plot of relative risks (RRs) and 95% confidence intervals (CIs) for the association between Mediterranean diet score (High vs. Low) and the incident risk of cognitive disorders by outcome type. MCI, mild cognitive impairment; AD, Alzheimer’s disease.

Figure 2 presents the forest plot of RRs (95% CIs) for the relationship of Mediterranean diet score (category of high vs. low) with the risk of developing cognitive disorders by type of outcomes. Mediterranean diet score was significantly associated with the incident risk of cognitive disorders in the random-effects meta-analysis of all 13 comparatives, and the pooled RR and 95% CI was 0.79 (0.70, 0.90), with no evidence of significant heterogeneity (I^2^ = 22%, P = 0.22). In the subgroup analysis, the pooled RRs and 95% CIs of Mediterranean diet score were 0.83 (0.75, 0.93) for MCI, 0.60 (0.48, 0.77) for AD, and 1.07 (0.81, 1.42) for dementia. In the sensitivity analysis, results showed that further exclusion of any single comparative in one turn did not significantly alter the pooled RR, with a range from 0.76 (0.65, 0.89) to 0.82 (0.73, 0.92). Exclusion of the study by Morris *et al*.^14^ did not significantly change the overall pooled analysis, and the corresponding RR (95% CI) was 0.82 (0.74, 0.91). As shown in Supplementary Figure 1(a), no publication bias was observed among the 13 comparatives, and the *p* values were 0.855 for Egger’s test, and 0.328 for Begg’s test.

**Figure 3:**
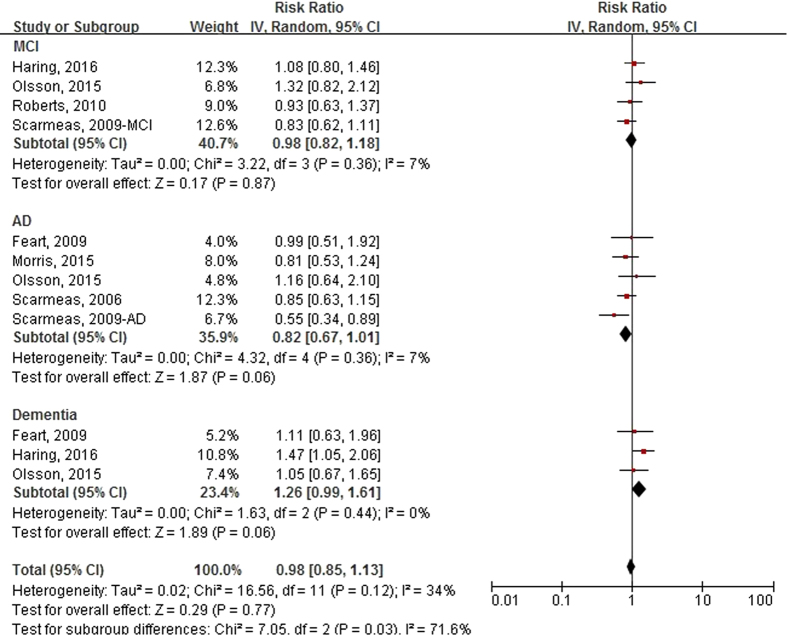
Forest plot of relative risks (RRs) and 95% confidence intervals (CIs) for the association between Mediterranean diet score (Median vs. Low) and the incident risk of cognitive disorders by outcome type. MCI, mild cognitive impairment; AD, Alzheimer’s disease.

Figure 3 shows the forest plot of RRs (95% CIs) for the association between Mediterranean diet score (category of median vs. low) and the risk of incident cognitive disorders. Mediterranean diet score was not significantly associated with the risk of developing cognitive disorders in the random-effects meta-analysis of all 12 comparatives, and the pooled RR (95% CI) was 0.98 (0.85, 1.13), with no evidence of significant heterogeneity (I^2^ = 34%, P = 0.12). Non-significant results were observed for the association between Mediterranean diet score and three types of cognitive disorders. Sensitivity analysis showed that the further exclusion of any single comparative did not significantly alter the overall combined RR, with a range from 0.97 (0.82, 1.14) to 1.06 (0.91, 1.23). Supplementary Figure 1(b) shows that no publication bias was found among the 12 comparatives (Egger’s test, P = 0.631; Begg’s test, P = 0.902).

**Figure 4:**
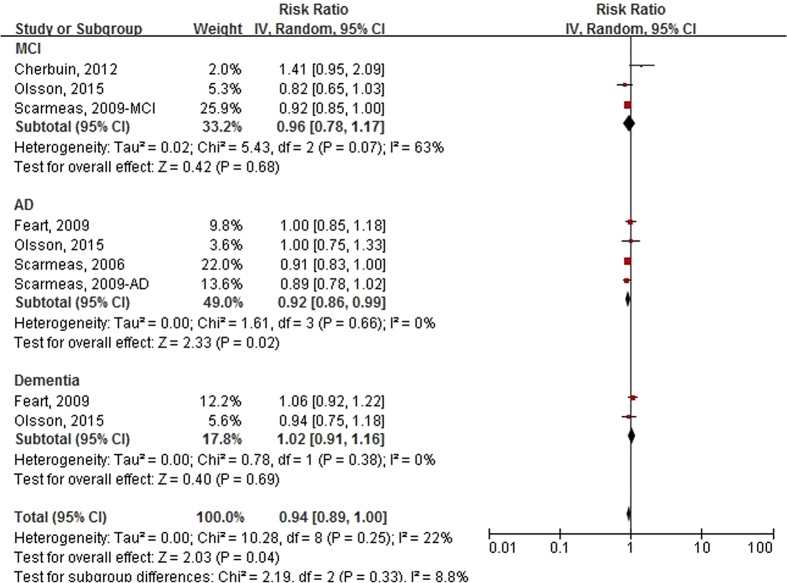
Forest plot of relative risks (RRs) and 95% confidence intervals (CIs) for the association between Mediterranean diet score (Continuous) and the incident risk of cognitive disorders by outcome type. MCI, mild cognitive impairment; AD, Alzheimer’s disease.

Figure 4 presents the forest plot of RRs (95% CIs) for the association between Mediterranean diet score (continuous variable) and the risk of incident cognitive disorders. Significant associations were observed for the cognitive disorders of 9 comparatives and AD of 4 comparatives, and the pooled RRs (95% CIs) were 0.94 (0.89, 1.00) and 0.92 (0.86, 0.99), respectively. There were no publication bias among the 9 comparatives (Supplementary Figure 1(c), Egger’s test, P = 0.348; Begg’s test, P = 0.211).

### Subgroup meta-analysis

As shown in Supplementary Table 3, stratified analyses by gender and by follow-up duration significantly affected the association between Mediterranean diet score (median vs. low) and the incident risk of cognitive disorders (P-value for difference < 0.05).

### Dose-response analysis

**Figure 5:**
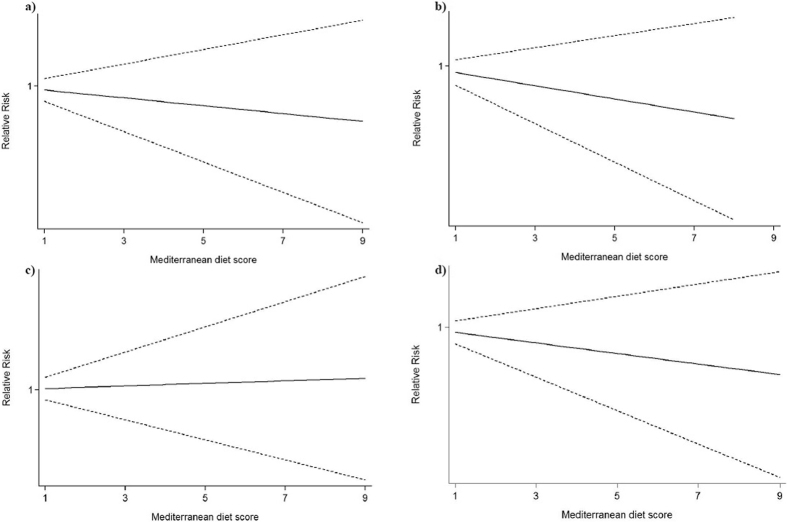
Dose-response association between Mediterranean diet score and cognitive disorders. (**a**) Mild cognitive impairment, (**b**) Alzheimer’s disease, (**c**) Dementia, (**d**) Cognitive disorders. Solid line, best-fitting restricted cubic spine; dotted line, 95% CI.

Seven studies with 12 comparisons reported more than 3 categories of Mediterranean diet score, and these comparatives were included in the overall dose-response analyses. The association between Mediterranean diet score and the incident risk of Dementia, AD and MCI were reported in 5, and 4 comparisons, respectively. Non-linear association between Mediterranean diet and the incident risk of cognitive disorders were non-significant for all types of cognitive disorders (all P for nonlinearity > 0.05). Under the linear hypothesis, there was a trend that higher Mediterranean diet score was associated with a decreased risk of incident cognitive disorders, but the result was not significant, and the summary RR (95% CI) was 0.97 (0.91, 1.03). The summary RRs (95% CIs) of the association between Mediterranean diet and the incident risk of MCI, AD and Dementia were 0.97 (0.89, 1.06), 0.94 (0.84, 1.06), and 1.01 (0.90, 1.15), respectively (Fig. 5).

## Discussion

The present systematic review and meta-analysis identified 9 cohort studies involving a total number of 34,168 participants. Compared with the lowest category of Mediterranean diet score, the pooled analysis showed that the highest Mediterranean diet score was inversely associated with the developing of cognitive disorders; however, Mediterranean diet score of the median category was not significantly associated with the incident risk of cognitive disorders. The dose-response analysis indicated a trend of a linear relationship of the Mediterranean diet score with the incident risk of cognitive disorders, but the association was not significant. In addition, there might be difference of the association between different gender and follow-up duration.

It is biologically plausible that Mediterranean diet is related to the decreased risk of the occurrence of cognitive disorders. First, a Mediterranean-like diet has been considered as a protective factor against many chronic diseases. By lowering the important risk factors and comorbidities of cardiovascular diseases, such as high blood pressure, dyslipidemia and diabetes, the proposed cardio-protective effect of Mediterranean diet may be an important pathway for the protective effect of Mediterranean diet on cognition^24^,^25^,^26^,^27^. The PREDIMED study conducted a randomized trial to evaluate the association between adherence to Mediterranean diet pattern and cardiovascular risk^28^, they found that the risk of stroke was significantly reduced in the two intervention groups. Hemorrhagic stroke is a known risk factor of dementia; as a result, adherence to Mediterranean diet is also likely to be associated with the lower risk of cognitive disorders. In fact, a randomized clinical trial has directly demonstrated that a Mediterranean-style supplemented diet was significantly improved the cognitive function in a Spanish older population^29^. Second, dietary component, such as fruit, vegetables, cereals, alcohol and unsaturated fatty acids in the Mediterranean diet have been demonstrated to be associated with a lower risk of cognitive decline^30^,^31^,^32^. As a mixture of these beneficial components, Mediterranean diet may also play a protective role against cognitive decline. Third, It is reported that adherence to Mediterranean diet was related to lower levels of C-reactive protein and interleukin^33^,^34^. The neuro-protective effects of Mediterranean diet may relate to its ability of reducing inflammation and oxidative stress, which are also linked to the pathophysiology of degenerative disease^35^,^36^.

The robustness of our finding was demonstrated in the sensitivity analysis. Only 1 included study did not adjust for the variable of ApoEε4 gene type in the multivariate statistical model, and the ApoEε4 gene is considered as an unequivocal risk factor of cognitive disorders^37^. Therefore, Mediterranean diet might be interpreted as an independent protective factor of cognitive disorders. The gender-difference of the association in the stratified study might be attributed to the non-significant result in the individual study. Both the study included only males^13^ and the study included only females^15^ reported a non-significant association between Mediterranean diet score and cognitive disorders. We speculated that the study population from the well-educated, middle- to high-income white women might dilute the association between Mediterranean diet and cognitive disorders^15^. Olive oil was a key component in the original Mediterranean diet score and has been demonstrated to have significantly improved the cognitive function in prospective studies^38^,^39^. The male participants in the study by Olsson *et al*. consumed relatively lower amount of olive oil compared with other studies, and thus this may reduce the beneficial role of Mediterranean diet in the prevention of cognitive disorders^15^. It is worth mention that the inverse association of Mediterranean diet and MCI was observed, but the association was not detected in the incident risk of dementia in the present study. We inferred that a Mediterranean-style diet may delay the progression of cognitive decline at the early stage of the cognitive impairment/decline. However, the Mediterranean diet would be unable to modify or delay the dementia onset preceding the clinical diagnosis.

Major strengths of the present systematic review and meta-analysis are the longitudinal design of the included articles. It is feasible to pool the effect sizes on account of the absence of statistical heterogeneity. Although several quantitative meta-analyses have reported the association between Mediterranean diet and cognitive disorders^9^,^10^,^11^,^12^, an additional 4 cohort studies with 25,864 participants were included in the present study compared with the last published meta-analysis in this topic. Furthermore, this is the first attempt to quantify the potential dose-response patterns of Mediterranean diet score on the risk of developing cognitive disorders. An approximately linear relationship of Mediterranean diet score and cognitive disorders was observed.

However, our study has limitations. First, articles provided the Mediterranean score were all included in the pooled analysis, and thus we included one study using other strategy to define the Mediterranean diet score (0–55 points). Although this study did not significantly change the overall pooled analysis in the sensitivity analysis, possible biases might have existed. Second, although Mediterranean diet score has been regarded as a reliable tool to measure the adherence of the Mediterranean-style diet^40^, measurement bias might have been given. Mediterranean diet has been evaluated in many different ways across studies, and these studies may use different versions to measure the individual component of the Mediterranean diet^41^. For instance, a low consumer from one study could be considered as high consumer in another study and vice versa, and this may have caused misclassification^42^. This could also explain the reason why we observed slightly different results from different regions. Detailed descriptions of the Mediterranean diet were poorly reported in the included studies, and thus this limited us to do further analysis to evaluate the effects of Mediterranean diet on cognitive disorders. Third, lifestyle and dietary habits were different among various regions. Changes in available year-round fruit, vegetables and other foods may have affected our results. Third, we extracted the data with the largest number of adjusted covariates in the multivariate statistical models; however, other unmeasured variables may also exist. Considering the complexity of the eating, lifestyle and environmental factors, it is difficult to identify the independent impact of a dietary pattern from other confounding variables. Fourth, the follow-up duration in most of the included studies was relatively short, and this may bring bias to our study. Finally, because of the observational nature of the present study, a causal association between Mediterranean diet and cognitive disorders cannot be established. Therefore, the pooled results of our study should be interpreted with caution. More conclusive evidence is needed to detect the causal effects of the Mediterranean diet on the occurrence of cognitive disorders.

## Conclusion

In summary, the results from the present updated systematic review and meta-analysis provide significant evidence of an inverse association between Mediterranean diet and the risk of developing cognitive disorders. Further studies of randomized controlled trials are warranted to confirm the observed association in different populations.

## Methods

### Literature Search

We conducted the present systematic review and meta-analysis according to standard criteria^43^,^44^. We searched the PubMed and the Embase from inception to August 13th, 2016 for records relevant to the Mediterranean diet and the risk of developing cognitive disorders with no language restriction. Our research terms included “Mediterranean”, “dementia”, “AD”, “Alzheimer*”, “aphronesia” and “cognitive*”, etc. (Supplementary Table 1). We manually searched the reference lists of the relevant original articles and systematic reviews in order to ascertain more eligible articles. When multiple publications from the same study were identified, the article with the longest duration of follow-up was included in the present analysis.

### Selection Criteria and Data Extraction

Two authors (Wu and Sun) performed the initial screen following the selection criteria independently. Duplicate records were excluded, and the title and abstract of each article was assessed independently for further assessment or exclusion. Any disagreements were resolved by discussion between the two authors.

The inclusion criteria were listed as follows: (1) studies reported the incident risk of developing cognitive disorders (mild cognitive impairment, cognitive decline, AD, and dementia) in participants with adherence to Mediterranean diet by using adjusted relative risks (RRs) or hazard ratios (HRs) or odds ratios (ORs) and their corresponding 95% confidence intervals (CIs); (2) adherence to the Mediterranean diet was defined according to the Mediterranean score. All articles provided the Mediterranean score were included in the pooled analysis, regardless of different category and different total points of the Mediterranean diet score. For instance, continuous, tertile and quintile category of Mediterranean score were all included in the present study. (3) prospective cohort design. Studies were excluded if: (1) data reported single nutrients or individual element of the Mediterranean diet; (2) the study subjects were not adults; (3) the article was a conference abstract.

The following information were extracted by Wu and Sun independently from each article: first author, year of publication, study location, sample size, gender and age of the study participants, number of incident cases, method of exposure and outcome measurements, type of outcome, follow-up duration, category of Mediterranean diet score, ORs or RRs or HRs with corresponding 95% CIs of the incident cognitive disorders for all categories of Mediterranean diet score with the largest number of adjusted covariates.

### Quality Assessment

We used the Newcastle-Ottawa quality scale (NOS)^45^ to assess the quality of the included articles. Higher score indicated higher study quality, and the scale was ranged between 0 to 9 points. The following three domains were assessed: the basis of the cohort selection (representativeness of the exposed cohort, selection of the non-exposed cohort, ascertainment of exposure to implants, and demonstration that outcome of interest was not present at start of study); the comparability of the cohort design and analysis (study controls for important and any additional covariates); and the adequacy of the exposure and outcome measurements (assessment of outcome, follow-up long enough for outcomes to occur, and adequacy of follow up).

### Statistical analysis

A generic inverse-variance method with random-effects model was used to pool the outcome data for Mediterranean diet score as categorical variables (medium vs. low, high vs. low) and continuous variable. Between-study heterogeneity was examined by the Q test and I^2^ statistic. I^2^ statistic higher than 50% was indicated as significant heterogeneity^46^. Additionally, we performed the stratified meta-analysis based on the pre-specified characteristics as follows: type of cognitive disorders (MCI, AD, dementia), study location (US, non-US), gender (male, female, both sexes), duration of follow-up (<5 years, ≥5 years), and exposure assessment method (FFQ, others). Sensitivity analysis was conducted to estimate the influence of a single article on the overall pooled analysis. Begg’s and Egger’s tests were used to test the potential publication bias^47^,^48^.

We further estimate the possible dose-response patterns of Mediterranean diet score on the risk of developing cognitive disorders. Generalized least-squares trend estimation (GLST) model was used to calculate the study-specific slopes^49^,^50^. For categories of the Mediterranean diet score that were open (e.g., 4–6 scores), we assigned the mean values as the corresponding score of Mediterranean diet. If a study did not use the 9-point scale of Mediterranean diet score, we converted the score to the 9-point scale. If the number of cases for each category was not reported, we used the RRs to obtain a summary estimate^51^. The dose-response results in the forest plots were presented for every one score increment in Mediterranean diet. A three-knot restricted cubic spline model was used to test for non-linearity hypothesis in the association. If the test for the non-linear association was not statistically significant, a GLST model without the restricted cubic spline was used to test the linear hypothesis.

All statistical analyses were conducted with the STATA software (version 12.0) and the Review Manager software (version 5.2). A two-tailed *P* value of less than 0.05 was considered as statistically significant.

## Additional Information

**How to cite this article:** Wu, L. and Sun, D. Adherence to Mediterranean diet and risk of developing cognitive disorders: An updated systematic review and meta-analysis of prospective cohort studies. *Sci. Rep.* **7**, 41317; doi: 10.1038/srep41317 (2017).

**Publisher's note:** Springer Nature remains neutral with regard to jurisdictional claims in published maps and institutional affiliations.

## Supplementary Material

Supplementary Materials

## Figures and Tables

**Table 1:** Characteristics of the included articles.

First author, Published year	Country	Participant selection	Follow-up (yrs)	Male (%)	Baseline age (yrs) (min-max)	Participants, No.	Mediterranean Diet score		Incident cognitive disorders			Adjustment^2^
							Method of assessment	Category	Method of ascertainment	Type	Case, No.	
Cherbuin, 2011	Australia	Cognitively normal participants	8	48.2	60–64	1,528	FFQ	Continuous	MCI	MMSE	10	1–10
Feart, 2009	France	Cognitively normal participants	4.1	37.0	65-	1,410	FFQ	Continuous, tertiles (0–9 scores)	Dementia; AD	DSM-IV; NINCDS-ADRDA	74; 50	2–9, 11–16
Haring, 2016	US	Participants without dementia	9.1	0.0	65–79	6,425	FFQ	Quintiles (0–9 scores)	MCI; Dementia	MMSE; DSM-IV	499; 390	1, 3–6, 8, 9, 17–23
Morris, 2015	US	Participants without AD	4.5	24.0	58–98	923	FFQ	Tertiles (0–55 scores)	AD	NINCDS-ADRDA	135	1–4, 6, 12, 23, 24
Olsson, 2015	Sweden	Participants without dementia	12	100.0	70-	1,038	7-d records	Continuous, tertiles (0–9 scores)	AD; Dementia; MCI	NINCDS-ADRDA; DSM-IV; MMSE	84; 143; 198	3, 4, 6, 12, 16, 25
Roberts, 2010	US	Participants without dementia	2.2	53	70–89	1,233	Health Habits and History Questionnaire	Tertiles (0–9 scores)	MCI	Standard criteria^1^	163	1–4, 7, 12, 26, 27
Scarmeas, 2006	US	Cognitively normal participants	4	32.0	65-	2,258	FFQ	Continuous, tertiles (0–9 scores)	AD	NINCDS-ADRDA	262	1–5,10, 16, 17, 20, 28, 29
Scarmeas, 2009-MCI	US	Cognitively normal participants	4.5	32.0	65-	1,393	FFQ	Continuous, tertiles (0–9 scores)	MCI	Standard criteria^1^	275	1–5,10, 16, 17, 28, 30
Scarmeas, 2009- AD	US	Participants with MCI	4.3	32.0	65-	482	FFQ	Continuous, Tertiles (0–9 scores)	AD	NINCDS-ADRDA	106	1–5,10, 16, 17, 28, 30
Tsivgoulis, 2013	US	Participants without MCI	4	43.0	45-	17,478	FFQ	Binary (0–9 scores)	MCI	Change between participants’ first and last scores on the 6-item Screener	1,248	1–3, 9, 17, 27, 31–33

AD, Alzheimer’s disease; MCI, mild cognitive impairment; FFQ, food frequency questionnaire; MMSE, Mini-Mental State Examination; DSM, Diagnostic and Statistical Manual of Mental Disorders; NINCDS-ADRDA, National Institute of Neurological and Communicative Disorders and Stroke-Alzheimer’s Disease and Related Disorders Association; CDR, Clinical Dementia Rating.

^1^Cognitive concern by others; impairment in 1 or more of the 4 cognitive domains from the cognitive testing battery; essentially normal functional activities from the CDR and Functional Activities Questionnaire; and absence of dementia.

^2^1 = age, 2 = sex, 3 = education, 4 = ApoEε4 gene, 5 = body mass index, 6 = physical activity, 7 = stroke, 8 = diabetes, 9 = hypertension, 10 = total caloric intake, 11 = marital status, 12 = total energy intake, 13 = taking 5 medications/d or more, 14 = Center, 15 = hypercholesterolemia, 16 = tobacco use, 17 = race, 18 = Women’s Health Initiative Hormone Trial randomization assignment, 19 = baseline MMSE score, 20 = smoking status, 21 = family income, 22 = depression, 23 = history of cardiovascular disease, 24 = participation in cognitively stimulating activities, 25 = living alone, 26 = coronary heart disease, 27 = depressive symptoms, 28 = cohort, 29 = comorbidity index, 30 = time between the first dietary assessment and the first cognitive assessment, 31 = environmental, 32 = vascular risk factors, 33 = self-reported health status.
