# Supplementary material for: Adherence to Mediterranean diet and risk of developing cognitive disorders: An updated systematic review and meta-analysis of prospective cohort studies
Source: Sci Rep. 2017 Jan 23;7:41317. doi: 10.1038/srep41317 (PMC5256032; doi:10.1038/srep41317)
Supplement: Supplementary Materials [file srep41317-s1.doc]

**Adherence to Mediterranean diet and risk of developing cognitive disorders: An updated systematic review and meta-analysis of prospective cohort studies**

**Running title:** Mediterranean diet and cognitive disorders

Lei Wu ^1*^, Dali Sun ^2^

^1^ Department of Epidemiology, Institute of Geriatrics, Chinese People's Liberation Army General Hospital, Beijing, China.

^2^ Department of Nanomedicine, Houston Methodist Research Institute, Houston, United States.

***** Author to whom correspondence should be addressed; E-Mail: [wlyg0118@163.com](mailto:wlyg0118@163.com), [wulei@301.cn](mailto:wulei@301.cn); Tel.: +86-10-66876415; Fax: +86-10-68219351.

| **Supplementary Table 1.** Search strategy |
| --- |
| Source: PubMed (Searched on: August 13th, 2016) |
| #1 mediterranean [Title/Abstract]  #2 dementia[title/abstract]  #3 AD[title/abstract]  #4 Alzheimer*[title/abstract]  #5 aphronesia[title/abstract]  #6 cognitive*[title/abstract]  #7 prospective[Title/Abstract] OR cohort[Title/Abstract] OR “follow up”[Title/Abstract] OR following[Title/Abstract] OR longitudinal[Title/Abstract] OR incidence[Title/Abstract] OR "prospective studies"[Mesh] OR "cohort studies"[Mesh] OR "longitudinal studies"[Mesh]  #8 #2 OR #3 OR #4 OR #5 OR #6  #9 #1 AND #7 AND #8 |
| Source: Embase (Searched on: August 13th, 2016) |
| #1 mediterranean:ti,ab  #2 dementia:ti,ab  #3 AD:ti,ab  #4 Alzheimer*:ti,ab  #5 aphronesia:ti,ab  #6 cognitive*:ti,ab  #7 ('prospective' OR 'cohort' OR 'follow up' OR 'longitudinal' OR 'incidence'):ti,ab  #8 #2 OR #3 OR #4 OR #5 OR #6  #7 #1 AND #7 AND #8 |

| **Supplementary Table 2.** The Newcastle-Ottawa Scale used to grade the quality of each study (maximum=9 stars) | | | | |
| --- | --- | --- | --- | --- |
| First author, published year | Selection | Comparability | Outcome | Total |
| Cherbuin, 2012 | **** | ** | *** | ********* |
| Feart, 2009 | **** | ** | ** | ******** |
| Haring, 2016 | **** | ** | *** | ********* |
| Morris, 2015 | **** | ** | ** | ******** |
| Olsson, 2015 | *** | ** | *** | ******** |
| Roberts, 2010 | *** | ** | ** | ******* |
| Scarmeas, 2006 | **** | ** | ** | ******** |
| Scarmeas, 2009 | **** | ** | ** | ******** |
| Tsivgoulis, 2013 | **** | ** | ** | ******** |
| Selection column includes four items: (1) representativeness of the exposed cohort, (2) selection of the non-exposed cohort, (3) ascertainment of exposure to implants, and (4) demonstration that outcome of interest was not present at start of study. Comparability column includes two items: (1) study controls for important and (2) any additional covariates. Outcome column includes three items: (1) assessment of outcome, (2) follow-up long enough for outcomes to occur, and (3) adequacy of follow up. | | | | |

| **Supplementary Table 3.** Stratified analysis of the association between Mediterranean diet score and the incident risk of cognitive disorders. | | | | | | |
| --- | --- | --- | --- | --- | --- | --- |
| Outcome | Mediterranean diet score (high vs. low) | | Mediterranean diet score (median vs. low) | | Mediterranean diet score (continuous) | |
|  | Comparisons, No. | RR (95% CI) | Comparisons, No. | RR (95% CI) | Comparisons, No. | RR (95% CI) |
| Total | 13 | 0.79 (0.70, 0.90) | 12 | 0.98 (0.85, 1.13) | 9 | 0.94 (0.89, 1.00) |
| Study location |  |  |  |  |  |  |
| US | 8 | 0.76 (0.64, 0.90) | 7 | 0.92 (0.75, 1.13) | 3 | 0.91 (0.87, 0.96) |
| Non-US | 5 | 0.89 (0.64, 1.21) | 5 | 1.13 (0.89, 1.44) | 6 | 1.00 (0.91, 1.11) |
| P-value for difference |  | 0.471 |  | 0.254 |  | 0.094 |
| Gender |  |  |  |  |  |  |
| Male | 3 | 0.80 (0.53, 1.22) | 3 | 1.17 (0.87, 1.56) | 3 | 0.91 (0.79, 1.05) |
| Female | 2 | 0.95 (0.70, 1.31) | 2 | 1.25 (0.92, 1.69) | - | - |
| Both sexes | 8 | 0.74 (0.62, 0.87) | 7 | 0.84 (0.72, 1.97) | 6 | 0.95 (0.89, 1.02) |
| P-value for difference |  | 0.354 |  | 0.023 |  | 0.736 |
| Duration of follow-up (yrs) |  |  |  |  |  |  |
| <5 | 8 | 0.74 (0.62, 0.87) | 7 | 0.84 (0.72, 1.97) | 5 | 0.94 (0.89, 0.99) |
| ≥5 | 5 | 0.91 (0.74, 1.12) | 5 | 1.21 (1.01, 1.45) | 4 | 0.98 (0.81, 1.18) |
| P-value for difference |  | 0.215 |  | 0.011 |  | 0.798 |
| Exposure assessment method |  |  |  |  |  |  |
| FFQ | 9 | 0.78 (0.66, 0.92) | 8 | 0.94 (0.77, 1.13) | 6 | 0.95 (0.89, 1.02) |
| Others | 4 | 0.79 (0.59, 1.07) | 4 | 1.08 (0.85, 1.36) | 3 | 0.91 (0.79, 1.05) |
| P-value for difference |  | 0.950 |  | 0.415 |  | 0.736 |
| FFQ, food-frequency questionnaire; RR, relative risk; CI, confidence interval | | | | | | |

| **Supplementary Table 4.** Excluded studies | |
| --- | --- |
| Reference | Reason For Exclusion |
| [Gardener S](http://www.ncbi.nlm.nih.gov/pubmed/?term=Gardener S%5BAuthor%5D&cauthor=true&cauthor_uid=23032941), [Gu Y](http://www.ncbi.nlm.nih.gov/pubmed/?term=Gu Y%5BAuthor%5D&cauthor=true&cauthor_uid=23032941), [Rainey-Smith SR](http://www.ncbi.nlm.nih.gov/pubmed/?term=Rainey-Smith SR%5BAuthor%5D&cauthor=true&cauthor_uid=23032941), [Keogh JB](http://www.ncbi.nlm.nih.gov/pubmed/?term=Keogh JB%5BAuthor%5D&cauthor=true&cauthor_uid=23032941), [Clifton PM](http://www.ncbi.nlm.nih.gov/pubmed/?term=Clifton PM%5BAuthor%5D&cauthor=true&cauthor_uid=23032941), [Mathieson SL](http://www.ncbi.nlm.nih.gov/pubmed/?term=Mathieson SL%5BAuthor%5D&cauthor=true&cauthor_uid=23032941), [Taddei K](http://www.ncbi.nlm.nih.gov/pubmed/?term=Taddei K%5BAuthor%5D&cauthor=true&cauthor_uid=23032941), [Mondal A](http://www.ncbi.nlm.nih.gov/pubmed/?term=Mondal A%5BAuthor%5D&cauthor=true&cauthor_uid=23032941), [Ward VK](http://www.ncbi.nlm.nih.gov/pubmed/?term=Ward VK%5BAuthor%5D&cauthor=true&cauthor_uid=23032941), [Scarmeas N](http://www.ncbi.nlm.nih.gov/pubmed/?term=Scarmeas N%5BAuthor%5D&cauthor=true&cauthor_uid=23032941), [Barnes M](http://www.ncbi.nlm.nih.gov/pubmed/?term=Barnes M%5BAuthor%5D&cauthor=true&cauthor_uid=23032941), [Ellis KA](http://www.ncbi.nlm.nih.gov/pubmed/?term=Ellis KA%5BAuthor%5D&cauthor=true&cauthor_uid=23032941), [Head R](http://www.ncbi.nlm.nih.gov/pubmed/?term=Head R%5BAuthor%5D&cauthor=true&cauthor_uid=23032941), [Masters CL](http://www.ncbi.nlm.nih.gov/pubmed/?term=Masters CL%5BAuthor%5D&cauthor=true&cauthor_uid=23032941), [Ames D](http://www.ncbi.nlm.nih.gov/pubmed/?term=Ames D%5BAuthor%5D&cauthor=true&cauthor_uid=23032941), [Macaulay SL](http://www.ncbi.nlm.nih.gov/pubmed/?term=Macaulay SL%5BAuthor%5D&cauthor=true&cauthor_uid=23032941), [Rowe CC](http://www.ncbi.nlm.nih.gov/pubmed/?term=Rowe CC%5BAuthor%5D&cauthor=true&cauthor_uid=23032941), [Szoeke C](http://www.ncbi.nlm.nih.gov/pubmed/?term=Szoeke C%5BAuthor%5D&cauthor=true&cauthor_uid=23032941), [Martins RN](http://www.ncbi.nlm.nih.gov/pubmed/?term=Martins RN%5BAuthor%5D&cauthor=true&cauthor_uid=23032941); [AIBL Research Group](http://www.ncbi.nlm.nih.gov/pubmed/?term=AIBL Research Group%5BCorporate Author%5D). Adherence to a Mediterranean diet and Alzheimer’s disease risk in an Australian population. [Transl Psychiatry.](http://www.ncbi.nlm.nih.gov/pubmed/?term=Adherence+to+a+Mediterranean+diet+and+Alzheimer’s+disease+risk+in+an+Australian+population) 2012;2:e164 | Non RCT: Cross-sectional study |
| [Féart C](http://www.ncbi.nlm.nih.gov/pubmed/?term=Féart C%5BAuthor%5D&cauthor=true&cauthor_uid=21303575), [Torrès MJ](http://www.ncbi.nlm.nih.gov/pubmed/?term=Torrès MJ%5BAuthor%5D&cauthor=true&cauthor_uid=21303575), [Samieri C](http://www.ncbi.nlm.nih.gov/pubmed/?term=Samieri C%5BAuthor%5D&cauthor=true&cauthor_uid=21303575), [Jutand MA](http://www.ncbi.nlm.nih.gov/pubmed/?term=Jutand MA%5BAuthor%5D&cauthor=true&cauthor_uid=21303575), [Peuchant E](http://www.ncbi.nlm.nih.gov/pubmed/?term=Peuchant E%5BAuthor%5D&cauthor=true&cauthor_uid=21303575), [Simopoulos AP](http://www.ncbi.nlm.nih.gov/pubmed/?term=Simopoulos AP%5BAuthor%5D&cauthor=true&cauthor_uid=21303575), [Barberger-Gateau P](http://www.ncbi.nlm.nih.gov/pubmed/?term=Barberger-Gateau P%5BAuthor%5D&cauthor=true&cauthor_uid=21303575). Adherence to a Mediterranean diet and plasma fatty acids: data from the Bordeaux sample of the three-city study. [Br J Nutr.](http://www.ncbi.nlm.nih.gov/pubmed/?term=Adherence+to+a+Mediterranean+diet+and+plasma+fatty+acids%3A+data+from+the+Bordeaux+sample+of+the+three-city+study) 2011;106(1):149-58. | Non RCT: Cross-sectional study |
| [Tangney CC](http://www.ncbi.nlm.nih.gov/pubmed/?term=Tangney CC%5BAuthor%5D&cauthor=true&cauthor_uid=21177796), [Kwasny MJ](http://www.ncbi.nlm.nih.gov/pubmed/?term=Kwasny MJ%5BAuthor%5D&cauthor=true&cauthor_uid=21177796), [Li H](http://www.ncbi.nlm.nih.gov/pubmed/?term=Li H%5BAuthor%5D&cauthor=true&cauthor_uid=21177796), [Wilson RS](http://www.ncbi.nlm.nih.gov/pubmed/?term=Wilson RS%5BAuthor%5D&cauthor=true&cauthor_uid=21177796), [Evans DA](http://www.ncbi.nlm.nih.gov/pubmed/?term=Evans DA%5BAuthor%5D&cauthor=true&cauthor_uid=21177796), [Morris MC](http://www.ncbi.nlm.nih.gov/pubmed/?term=Morris MC%5BAuthor%5D&cauthor=true&cauthor_uid=21177796). Adherence to a Mediterranean-type dietary pattern and cognitive decline in a community population. [Am J Clin Nutr.](http://www.ncbi.nlm.nih.gov/pubmed/?term=Adherence+to+a+Mediterranean-type+dietary+pattern+and+cognitive+decline+in+a+community+population.) 2011;93(3):601-7. | No data able to be extracted. Cognitive disorders not defined or measured. |
| [Koyama A](http://www.ncbi.nlm.nih.gov/pubmed/?term=Koyama A%5BAuthor%5D&cauthor=true&cauthor_uid=24994847), [Houston DK](http://www.ncbi.nlm.nih.gov/pubmed/?term=Houston DK%5BAuthor%5D&cauthor=true&cauthor_uid=24994847), [Simonsick EM](http://www.ncbi.nlm.nih.gov/pubmed/?term=Simonsick EM%5BAuthor%5D&cauthor=true&cauthor_uid=24994847), [Lee JS](http://www.ncbi.nlm.nih.gov/pubmed/?term=Lee JS%5BAuthor%5D&cauthor=true&cauthor_uid=24994847), [Ayonayon HN](http://www.ncbi.nlm.nih.gov/pubmed/?term=Ayonayon HN%5BAuthor%5D&cauthor=true&cauthor_uid=24994847), [Shahar DR](http://www.ncbi.nlm.nih.gov/pubmed/?term=Shahar DR%5BAuthor%5D&cauthor=true&cauthor_uid=24994847), [Rosano C](http://www.ncbi.nlm.nih.gov/pubmed/?term=Rosano C%5BAuthor%5D&cauthor=true&cauthor_uid=24994847), [Satterfield S](http://www.ncbi.nlm.nih.gov/pubmed/?term=Satterfield S%5BAuthor%5D&cauthor=true&cauthor_uid=24994847), [Yaffe K](http://www.ncbi.nlm.nih.gov/pubmed/?term=Yaffe K%5BAuthor%5D&cauthor=true&cauthor_uid=24994847). Association between the Mediterranean diet and cognitive decline in a biracial population. [J Gerontol A Biol Sci Med Sci.](http://www.ncbi.nlm.nih.gov/pubmed/?term=Association+between+the+Mediterranean+diet+and++cognitive+decline+in+a+biracial+population) 2015;70(3):354-9. | No data able to be extracted. Cognitive disorders not defined or measured. |
| [Qin B](http://www.ncbi.nlm.nih.gov/pubmed/?term=Qin B%5BAuthor%5D&cauthor=true&cauthor_uid=26133024), [Adair LS](http://www.ncbi.nlm.nih.gov/pubmed/?term=Adair LS%5BAuthor%5D&cauthor=true&cauthor_uid=26133024), [Plassman BL](http://www.ncbi.nlm.nih.gov/pubmed/?term=Plassman BL%5BAuthor%5D&cauthor=true&cauthor_uid=26133024), [Batis C](http://www.ncbi.nlm.nih.gov/pubmed/?term=Batis C%5BAuthor%5D&cauthor=true&cauthor_uid=26133024), [Edwards LJ](http://www.ncbi.nlm.nih.gov/pubmed/?term=Edwards LJ%5BAuthor%5D&cauthor=true&cauthor_uid=26133024), [Popkin BM](http://www.ncbi.nlm.nih.gov/pubmed/?term=Popkin BM%5BAuthor%5D&cauthor=true&cauthor_uid=26133024), [Mendez MA](http://www.ncbi.nlm.nih.gov/pubmed/?term=Mendez MA%5BAuthor%5D&cauthor=true&cauthor_uid=26133024). Dietary patterns and cognitive decline among Chinese older adults. [Epidemiology.](http://www.ncbi.nlm.nih.gov/pubmed/26133024) 2015;26(5):758-68. | No data able to be extracted. Cognitive disorders not defined or measured. |
| [Gardener SL](http://www.ncbi.nlm.nih.gov/pubmed/?term=Gardener SL%5BAuthor%5D&cauthor=true&cauthor_uid=25070537), [Rainey-Smith SR](http://www.ncbi.nlm.nih.gov/pubmed/?term=Rainey-Smith SR%5BAuthor%5D&cauthor=true&cauthor_uid=25070537), [Barnes MB](http://www.ncbi.nlm.nih.gov/pubmed/?term=Barnes MB%5BAuthor%5D&cauthor=true&cauthor_uid=25070537), [Sohrabi HR](http://www.ncbi.nlm.nih.gov/pubmed/?term=Sohrabi HR%5BAuthor%5D&cauthor=true&cauthor_uid=25070537), [Weinborn M](http://www.ncbi.nlm.nih.gov/pubmed/?term=Weinborn M%5BAuthor%5D&cauthor=true&cauthor_uid=25070537), [Lim YY](http://www.ncbi.nlm.nih.gov/pubmed/?term=Lim YY%5BAuthor%5D&cauthor=true&cauthor_uid=25070537), [Harrington K](http://www.ncbi.nlm.nih.gov/pubmed/?term=Harrington K%5BAuthor%5D&cauthor=true&cauthor_uid=25070537), [Taddei K](http://www.ncbi.nlm.nih.gov/pubmed/?term=Taddei K%5BAuthor%5D&cauthor=true&cauthor_uid=25070537), [Gu Y](http://www.ncbi.nlm.nih.gov/pubmed/?term=Gu Y%5BAuthor%5D&cauthor=true&cauthor_uid=25070537), [Rembach A](http://www.ncbi.nlm.nih.gov/pubmed/?term=Rembach A%5BAuthor%5D&cauthor=true&cauthor_uid=25070537), [Szoeke C](http://www.ncbi.nlm.nih.gov/pubmed/?term=Szoeke C%5BAuthor%5D&cauthor=true&cauthor_uid=25070537), [Ellis KA](http://www.ncbi.nlm.nih.gov/pubmed/?term=Ellis KA%5BAuthor%5D&cauthor=true&cauthor_uid=25070537), [Masters CL](http://www.ncbi.nlm.nih.gov/pubmed/?term=Masters CL%5BAuthor%5D&cauthor=true&cauthor_uid=25070537), [Macaulay SL](http://www.ncbi.nlm.nih.gov/pubmed/?term=Macaulay SL%5BAuthor%5D&cauthor=true&cauthor_uid=25070537), [Rowe CC](http://www.ncbi.nlm.nih.gov/pubmed/?term=Rowe CC%5BAuthor%5D&cauthor=true&cauthor_uid=25070537), [Ames D](http://www.ncbi.nlm.nih.gov/pubmed/?term=Ames D%5BAuthor%5D&cauthor=true&cauthor_uid=25070537), [Keogh JB](http://www.ncbi.nlm.nih.gov/pubmed/?term=Keogh JB%5BAuthor%5D&cauthor=true&cauthor_uid=25070537), [Scarmeas N](http://www.ncbi.nlm.nih.gov/pubmed/?term=Scarmeas N%5BAuthor%5D&cauthor=true&cauthor_uid=25070537), [Martins RN](http://www.ncbi.nlm.nih.gov/pubmed/?term=Martins RN%5BAuthor%5D&cauthor=true&cauthor_uid=25070537). Dietary patterns and cognitive decline in an Australian study of ageing. [Mol Psychiatry.](http://www.ncbi.nlm.nih.gov/pubmed/?term=Dietary+patterns+and+cognitive+decline+in+an+Australian+study+of+ageing) 2015;20(7):860-6. | No data able to be extracted. Cognitive disorders not defined or measured. |
| [Samieri C](http://www.ncbi.nlm.nih.gov/pubmed/?term=Samieri C%5BAuthor%5D&cauthor=true&cauthor_uid=23365105), [Okereke OI](http://www.ncbi.nlm.nih.gov/pubmed/?term=Okereke OI%5BAuthor%5D&cauthor=true&cauthor_uid=23365105), [E Devore E](http://www.ncbi.nlm.nih.gov/pubmed/?term=E Devore E%5BAuthor%5D&cauthor=true&cauthor_uid=23365105), [Grodstein F](http://www.ncbi.nlm.nih.gov/pubmed/?term=Grodstein F%5BAuthor%5D&cauthor=true&cauthor_uid=23365105). Long-term adherence to the Mediterranean diet is associated with overall cognitive status, but not cognitive decline, in women. [J Nutr.](http://www.ncbi.nlm.nih.gov/pubmed/?term=Long-term+adherence+to+the+Mediterranean+diet+is+associated+with+overall+cognitive+status%2C+but+not+cognitive+decline%2C+in+women) 2013;143(4):493-9. | No data able to be extracted. Cognitive disorders not defined or measured. |
| [Samieri C](http://www.ncbi.nlm.nih.gov/pubmed/?term=Samieri C%5BAuthor%5D&cauthor=true&cauthor_uid=23365105), [Okereke OI](http://www.ncbi.nlm.nih.gov/pubmed/?term=Okereke OI%5BAuthor%5D&cauthor=true&cauthor_uid=23365105), [E Devore E](http://www.ncbi.nlm.nih.gov/pubmed/?term=E Devore E%5BAuthor%5D&cauthor=true&cauthor_uid=23365105), [Grodstein F](http://www.ncbi.nlm.nih.gov/pubmed/?term=Grodstein F%5BAuthor%5D&cauthor=true&cauthor_uid=23365105). Mediterranean diet and cognitive decline in the nurses' health study. [J Nutr.](http://www.ncbi.nlm.nih.gov/pubmed/?term=Mediterranean+diet+and+cognitive+decline+in+the+nurses'+health+study) 2013;143(4):493-9. | No data able to be extracted. Cognitive disorders not defined or measured. |
| [Vercambre MN](http://www.ncbi.nlm.nih.gov/pubmed/?term=Vercambre MN%5BAuthor%5D&cauthor=true&cauthor_uid=22709809), [Grodstein F](http://www.ncbi.nlm.nih.gov/pubmed/?term=Grodstein F%5BAuthor%5D&cauthor=true&cauthor_uid=22709809), [Berr C](http://www.ncbi.nlm.nih.gov/pubmed/?term=Berr C%5BAuthor%5D&cauthor=true&cauthor_uid=22709809), [Kang JH](http://www.ncbi.nlm.nih.gov/pubmed/?term=Kang JH%5BAuthor%5D&cauthor=true&cauthor_uid=22709809). Mediterranean diet and cognitive decline in women with Cardiovascular disease or risk factors. [J Acad Nutr Diet.](http://www.ncbi.nlm.nih.gov/pubmed/22709809) 2012;112(6):816-23. | No data able to be extracted. Cognitive disorders not defined or measured. |
| [Kesse-Guyot E](http://www.ncbi.nlm.nih.gov/pubmed/?term=Kesse-Guyot E%5BAuthor%5D&cauthor=true&cauthor_uid=23283500), [Andreeva VA](http://www.ncbi.nlm.nih.gov/pubmed/?term=Andreeva VA%5BAuthor%5D&cauthor=true&cauthor_uid=23283500), [Lassale C](http://www.ncbi.nlm.nih.gov/pubmed/?term=Lassale C%5BAuthor%5D&cauthor=true&cauthor_uid=23283500), [Ferry M](http://www.ncbi.nlm.nih.gov/pubmed/?term=Ferry M%5BAuthor%5D&cauthor=true&cauthor_uid=23283500), [Jeandel C](http://www.ncbi.nlm.nih.gov/pubmed/?term=Jeandel C%5BAuthor%5D&cauthor=true&cauthor_uid=23283500), [Hercberg S](http://www.ncbi.nlm.nih.gov/pubmed/?term=Hercberg S%5BAuthor%5D&cauthor=true&cauthor_uid=23283500), [Galan P](http://www.ncbi.nlm.nih.gov/pubmed/?term=Galan P%5BAuthor%5D&cauthor=true&cauthor_uid=23283500); [SU.VI.MAX 2 Research Group](http://www.ncbi.nlm.nih.gov/pubmed/?term=SU.VI.MAX 2 Research Group%5BCorporate Author%5D). Mediterranean diet and cognitive function: a French study. [Am J Clin Nutr.](http://www.ncbi.nlm.nih.gov/pubmed/23283500) 2013;97(2):369-76. | No data able to be extracted. Cognitive disorders not defined or measured. |
| [Samieri C](http://www.ncbi.nlm.nih.gov/pubmed/?term=Samieri C%5BAuthor%5D&cauthor=true&cauthor_uid=23676264), [Grodstein F](http://www.ncbi.nlm.nih.gov/pubmed/?term=Grodstein F%5BAuthor%5D&cauthor=true&cauthor_uid=23676264), [Rosner BA](http://www.ncbi.nlm.nih.gov/pubmed/?term=Rosner BA%5BAuthor%5D&cauthor=true&cauthor_uid=23676264), [Kang JH](http://www.ncbi.nlm.nih.gov/pubmed/?term=Kang JH%5BAuthor%5D&cauthor=true&cauthor_uid=23676264), [Cook NR](http://www.ncbi.nlm.nih.gov/pubmed/?term=Cook NR%5BAuthor%5D&cauthor=true&cauthor_uid=23676264), [Manson JE](http://www.ncbi.nlm.nih.gov/pubmed/?term=Manson JE%5BAuthor%5D&cauthor=true&cauthor_uid=23676264), [Buring JE](http://www.ncbi.nlm.nih.gov/pubmed/?term=Buring JE%5BAuthor%5D&cauthor=true&cauthor_uid=23676264), [Willett WC](http://www.ncbi.nlm.nih.gov/pubmed/?term=Willett WC%5BAuthor%5D&cauthor=true&cauthor_uid=23676264), [Okereke OI](http://www.ncbi.nlm.nih.gov/pubmed/?term=Okereke OI%5BAuthor%5D&cauthor=true&cauthor_uid=23676264). Mediterranean diet and cognitive function in older age: results from the Women’s Health Study. [Epidemiology.](http://www.ncbi.nlm.nih.gov/pubmed/?term=Mediterranean+diet+and+cognitive+function+in+older+age%3A+results+from+the+Women’s+Health+Study) 2013;24(4):490-9. | No data able to be extracted. Cognitive disorders not defined or measured. |
| [Galbete C](http://www.ncbi.nlm.nih.gov/pubmed/?term=Galbete C%5BAuthor%5D&cauthor=true&cauthor_uid=25732216), [Toledo E](http://www.ncbi.nlm.nih.gov/pubmed/?term=Toledo E%5BAuthor%5D&cauthor=true&cauthor_uid=25732216), [Toledo JB](http://www.ncbi.nlm.nih.gov/pubmed/?term=Toledo JB%5BAuthor%5D&cauthor=true&cauthor_uid=25732216), [Bes-Rastrollo M](http://www.ncbi.nlm.nih.gov/pubmed/?term=Bes-Rastrollo M%5BAuthor%5D&cauthor=true&cauthor_uid=25732216), [Buil-Cosiales P](http://www.ncbi.nlm.nih.gov/pubmed/?term=Buil-Cosiales P%5BAuthor%5D&cauthor=true&cauthor_uid=25732216), [Marti A](http://www.ncbi.nlm.nih.gov/pubmed/?term=Marti A%5BAuthor%5D&cauthor=true&cauthor_uid=25732216), [Guillén-Grima F](http://www.ncbi.nlm.nih.gov/pubmed/?term=Guillén-Grima F%5BAuthor%5D&cauthor=true&cauthor_uid=25732216), [Martínez-González MA](http://www.ncbi.nlm.nih.gov/pubmed/?term=Martínez-González MA%5BAuthor%5D&cauthor=true&cauthor_uid=25732216). Mediterranean diet and cognitive function The sun project. [J Nutr Health Aging.](http://www.ncbi.nlm.nih.gov/pubmed/?term=Mediterranean+diet+and+cognitive+function+The+sun+project) 2015;19(3):305-12. | No data able to be extracted. Cognitive disorders not defined or measured. |
| [Wengreen H](http://www.ncbi.nlm.nih.gov/pubmed/?term=Wengreen H%5BAuthor%5D&cauthor=true&cauthor_uid=24047922), [Munger RG](http://www.ncbi.nlm.nih.gov/pubmed/?term=Munger RG%5BAuthor%5D&cauthor=true&cauthor_uid=24047922), [Cutler A](http://www.ncbi.nlm.nih.gov/pubmed/?term=Cutler A%5BAuthor%5D&cauthor=true&cauthor_uid=24047922), [Quach A](http://www.ncbi.nlm.nih.gov/pubmed/?term=Quach A%5BAuthor%5D&cauthor=true&cauthor_uid=24047922), [Bowles A](http://www.ncbi.nlm.nih.gov/pubmed/?term=Bowles A%5BAuthor%5D&cauthor=true&cauthor_uid=24047922), [Corcoran C](http://www.ncbi.nlm.nih.gov/pubmed/?term=Corcoran C%5BAuthor%5D&cauthor=true&cauthor_uid=24047922), [Tschanz JT](http://www.ncbi.nlm.nih.gov/pubmed/?term=Tschanz JT%5BAuthor%5D&cauthor=true&cauthor_uid=24047922), [Norton MC](http://www.ncbi.nlm.nih.gov/pubmed/?term=Norton MC%5BAuthor%5D&cauthor=true&cauthor_uid=24047922), [Welsh-Bohmer KA](http://www.ncbi.nlm.nih.gov/pubmed/?term=Welsh-Bohmer KA%5BAuthor%5D&cauthor=true&cauthor_uid=24047922). Prospective study of dietary approaches to stop hypertension-and Mediterranean-style dietary patterns and age-related cognitive change: the Cache County Study on Memory, Health and Aging. [Am J Clin Nutr.](http://www.ncbi.nlm.nih.gov/pubmed/?term=Prospective+study+of+dietary+approaches+to+stop+hypertension-and+Mediterranean-style+dietary+patterns+and+age-related+cognitive+change%3A+the+Cache+County+Study+on+Memory%2C+Health+and+Aging) 2013;98(5):1263-71. | No data able to be extracted. Cognitive disorders not defined or measured. |
| [Tangney CC](http://www.ncbi.nlm.nih.gov/pubmed/?term=Tangney CC%5BAuthor%5D&cauthor=true&cauthor_uid=25230996), [Li H](http://www.ncbi.nlm.nih.gov/pubmed/?term=Li H%5BAuthor%5D&cauthor=true&cauthor_uid=25230996), [Wang Y](http://www.ncbi.nlm.nih.gov/pubmed/?term=Wang Y%5BAuthor%5D&cauthor=true&cauthor_uid=25230996), [Barnes L](http://www.ncbi.nlm.nih.gov/pubmed/?term=Barnes L%5BAuthor%5D&cauthor=true&cauthor_uid=25230996), [Schneider JA](http://www.ncbi.nlm.nih.gov/pubmed/?term=Schneider JA%5BAuthor%5D&cauthor=true&cauthor_uid=25230996), [Bennett DA](http://www.ncbi.nlm.nih.gov/pubmed/?term=Bennett DA%5BAuthor%5D&cauthor=true&cauthor_uid=25230996), [Morris MC](http://www.ncbi.nlm.nih.gov/pubmed/?term=Morris MC%5BAuthor%5D&cauthor=true&cauthor_uid=25230996). Relation of DASH- and Mediterranean-like dietary patterns to cognitive decline in older persons. [Neurology.](http://www.ncbi.nlm.nih.gov/pubmed/?term=Relation+of+DASH-+and+Mediterranean-like+dietary+patterns+to+cognitive+decline+in+older+persons) 2014;83(16):1410-6. | No data able to be extracted. Cognitive disorders not defined or measured. |
| [Cherbuin](http://xueshu.baidu.com/s?wd=author%3A(Nicolas Cherbuin) &tn=SE_baiduxueshu_c1gjeupa&ie=utf-8&sc_f_para=sc_hilight%3Dperson) N,  [Kumar](http://xueshu.baidu.com/s?wd=author%3A(Rajeev Kumar) &tn=SE_baiduxueshu_c1gjeupa&ie=utf-8&sc_f_para=sc_hilight%3Dperson) R, [Anstey](http://xueshu.baidu.com/s?wd=author%3A(Kaarin Anstey) &tn=SE_baiduxueshu_c1gjeupa&ie=utf-8&sc_f_para=sc_hilight%3Dperson) K. Caloric intake, but not the Mediterranean diet, is associated with cognition and mild cognitive impairment. Alzheimers & Dementia. 2011; 7(4):S691 | Conference abstract, duplicate cohort of Cherbuin, et al. 2011. |
| [Scarmeas N](http://www.ncbi.nlm.nih.gov/pubmed/?term=Scarmeas N%5BAuthor%5D&cauthor=true&cauthor_uid=19671904), [Luchsinger JA](http://www.ncbi.nlm.nih.gov/pubmed/?term=Luchsinger JA%5BAuthor%5D&cauthor=true&cauthor_uid=19671904), [Schupf N](http://www.ncbi.nlm.nih.gov/pubmed/?term=Schupf N%5BAuthor%5D&cauthor=true&cauthor_uid=19671904), [Brickman AM](http://www.ncbi.nlm.nih.gov/pubmed/?term=Brickman AM%5BAuthor%5D&cauthor=true&cauthor_uid=19671904), [Cosentino S](http://www.ncbi.nlm.nih.gov/pubmed/?term=Cosentino S%5BAuthor%5D&cauthor=true&cauthor_uid=19671904), [Tang MX](http://www.ncbi.nlm.nih.gov/pubmed/?term=Tang MX%5BAuthor%5D&cauthor=true&cauthor_uid=19671904), [Stern Y](http://www.ncbi.nlm.nih.gov/pubmed/?term=Stern Y%5BAuthor%5D&cauthor=true&cauthor_uid=19671904). Physical activity, diet, and risk of Alzheimer disease. [JAMA.](http://www.ncbi.nlm.nih.gov/pubmed/19671904) 2009;302(6):627-37. | Duplicate cohort of Scarmeas, et al. 2009. |
| Olsson E, Karlström B, [Kilander L](http://fou.nu/is/sverige/user/html/70641), Byberg L, [Cederholm T](http://fou.nu/is/sverige/user/html/7782), Sjögren P. [Dietary patterns and cognitive dysfunction in a prospective study of 70-year-old swedish men.](http://fou.nu/is/sverige/user/publication?ref=2221161) Ann Nutr Metab. 2013:63(Suppl.1):862. | Conference abstract, duplicate cohort of Olsson, 2015 |

**a)**

**b)**

**c)**

**Supplementary Figure 1.** Funnel plot for the analysis of category of Mediterranean diet score and the incident risk of cognitive disorders. a) High vs. low, b) Median vs. low, c) Continuous.
